# Pancreas-guided C-shaped surgical procedure: a safer and more efficient procedure for laparoscopic left hemicolectomy in obese patients

**DOI:** 10.1007/s13304-025-02071-x

**Published:** 2025-01-18

**Authors:** Huaqi Zhang, Sen Wang, Zhensheng Chen, Tedong Luo, Jinpeng Cao, Zhicheng Li, Yong Ji

**Affiliations:** Department of Gastrointestinal Surgery, The First People’s Hospital of Foshan, No. 81 Lingnan Avenue North, Foshan, China

**Keywords:** Laparoscopic left hemicolectomy, Obese patients, Splenic flexure, Surgical procedure, Left hemicolon cancer

## Abstract

The surgical risk is higher for obese patients undergoing laparoscopic left hemicolectomy. To enhance the surgical safety and efficacy for obese patients, we have innovatively integrated the advantages of various surgical approaches to modify a pancreas-guided C-shaped surgical procedure. The safety and quality were assessed through a retrospective analysis. Colon cancer patients who underwent laparoscopic left hemicolectomy were categorized into two groups, C-shaped group and Medial-to-lateral group. Baseline data, operative safety indices, operative quality indices and learning curve were subjected to statistical analysis. The complete mesocolic excision rate and R0 resection rate were 100% in both groups. In terms of surgical safety, C-shaped group experienced significantly less blood loss (50(20) mL vs. 50(50) mL, *p* = 0.002), shorter total operative time (252.65 ± 50.43 min vs. 280.12 ± 70.45 min, *p* = 0.004) and no organ damage occurred. All patients were classified into four BMI grades (I: BMI < 18.5 kg/m^2^; II: 18.5 ≤ BMI < 24 kg/m^2^; III: 24 ≤ BMI < 28 kg/m^2^; IV: BMI ≥ 28 kg/m^2^). The total operative time and estimated blood loss were significantly lower in obese patients (BMI grade III and IV) of C-shaped group. In addition, intra-group analysis further confirmed that this modified surgical technique could effectively enhance safety and efficiency for obese patients. Learning curve analysis revealed a significant reduction in total operative time after the completion of 20 surgeries. Utilization of the pancreas-guided C-shaped surgical procedure in obese patients ensures reliable surgical outcomes and significantly increases safety and efficiency. In addition, it is easier to learn and master.

**Supplementary Information:**

The online version contains supplementary material available at 10.1007/s13304-025-02071-x.

## Introduction

With the increasing emphasis on minimally invasive surgery, laparoscopic left hemicolectomy has become the most commonly utilized method for left hemicolectomy [1, 2]. The surgical risk and difficulty are frequently heightened in obese patients due to several factors. These include the presence of hypertrophic mesentery, unclear anatomic planes, and limited intra-abdominal space. Consequently, these factors contribute to increased challenges during splenic flexure mobilization and lymph node dissection, leading to a higher risk of bleeding, prolonged operation time, and an elevated likelihood of inadvertent injury to blood vessels and organs [3, 4]. Therefore, it is imperative to employ a safer and more effective surgical method for obese patients undergoing laparoscopic left hemicolectomy.

The splenic flexure mobilization poses a significant challenge in laparoscopic left hemicolectomy. The primary challenge involves the avoidance of pancreatic and splenic injuries, as well as the protection of crucial blood vessels, while simultaneously achieving a complete mesocolic excision. The medial-to-lateral approach is extensively utilized in surgical procedures [5, 6]. However, this approach has drawbacks in clinical application, such as potential entry into the retro-pancreatic space, leading to pancreatic injury during surgery, which is rare but fatal [7]. Many novel approaches, such as superior-inferior approach [8], cranial-to-caudal approach [9, 10], complete medial approach [11], “four-step” method [12] and bursa omentalis approach [13], have been implemented in clinical practice to overcome these shortcomings, proving to be superior in some respects. However, the clinical efficacy of these surgical approaches in obese patients have not been sufficiently evaluated.

Sufficient clinical evidence has demonstrated that utilizing the Treitz’s ligament and IMV (inferior mesenteric vein) as anatomic landmarks enables a easier and more precise access to Toldt’s space, thereby avoiding pancreatic injury associated with entry into the posterior pancreatic space [14, 15]. Simultaneously, upon entering the anterior pancreatic space through Toldt’s space, mobilization of the splenic flexure guided by pancreas can effectively minimize both pancreatic and spleen injuries, consequently enhancing surgical safety [8–13]. We use medial approach as the starting step for the surgery and optimized the overall surgical process, ultimately modifying a pancreas-guided C-shaped surgical procedure for obese patients. By implementing a more cohesive surgical procedure, we can increase surgical efficiency while minimizing the risk of inadvertent injury to blood vessels and organs. To validate its applicability in laparoscopic left hemicolectomy, we conducted a retrospective analysis to evaluate the surgical quality and safety of this innovative surgical procedure.

## Patients and methods

### Patients

We conducted a retrospective analysis of the clinical data from patients who underwent laparoscopic left hemicolectomy between 2013 and 2024. In this study, patients aged 18–90 years, regardless of sex, were preoperatively evaluated through colonoscopy and pathologic examination to confirm the diagnosis of malignant colon tumors. All surgeries were performed by the same group of surgeons. Patients who converted to open surgery, patients who underwent emergency surgery for intestinal obstruction or perforation, and patients who underwent palliative surgery were not included in the study. According to the surgical procedure used, all patients were categorized into either the C-shaped group or the Medial-to-lateral group. Our statistical analysis included patient demographic data, such as sex, age, BMI, tumor invasion status, tumor stage, and tumor location. In addition, we examined surgical safety indices, including total operative time, estimated blood loss, time to first flatus, time to first semi-fluid diet, length of postoperative hospital stay, and incidence of postoperative complications. Furthermore, we assessed surgical quality indices including the number of retrieved lymph nodes, the R0 resection rate, and the CME (complete mesocolic excision) rate. Finally, we analyzed the learning curve of the same surgeon performing these surgeries. Before surgery commenced for each patient, a detailed explanation of the surgical methods and associated risks was provided to ensure informed consent. The retrospective nature of our study has no impact on the treatment plan and outcomes for patients, and due to difficulties in contacting some patients, the exemption from informed consent was approved. This study received approval from the Ethics Committee of the First People’s Hospital of Foshan and adhered strictly to all requirements outlined in the Declaration of Helsinki.

### Surgical techniques

#### C-shaped group

**Fig. 1:**
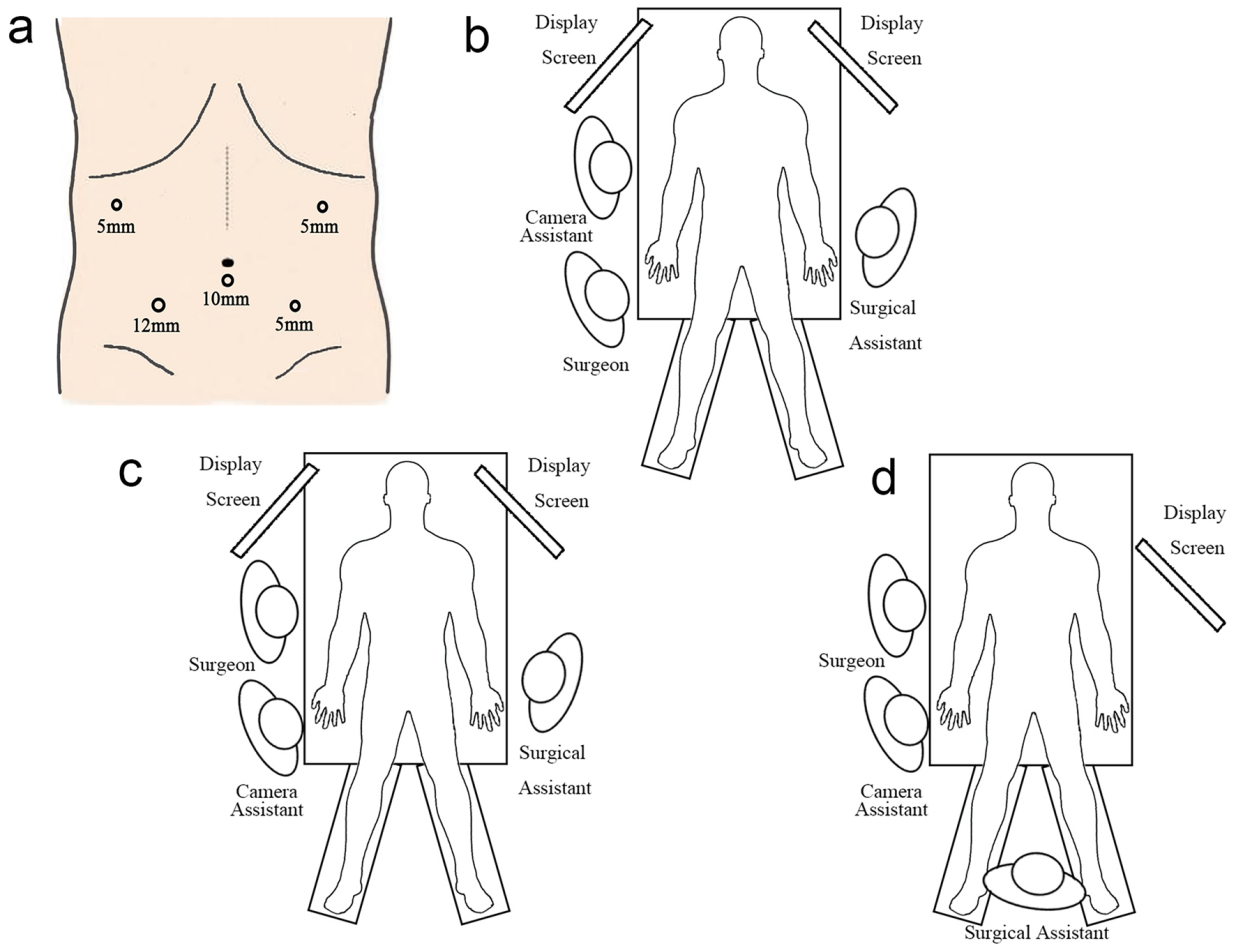
Preparation for surgery. **a** Recommended surgical incision. **b** Recommended surgeons’ position during step 1,4,5,6. **c** Recommended surgeons’ position during step 2,3. **d** Recommended surgeons’ position during step 7

**Fig. 2:**
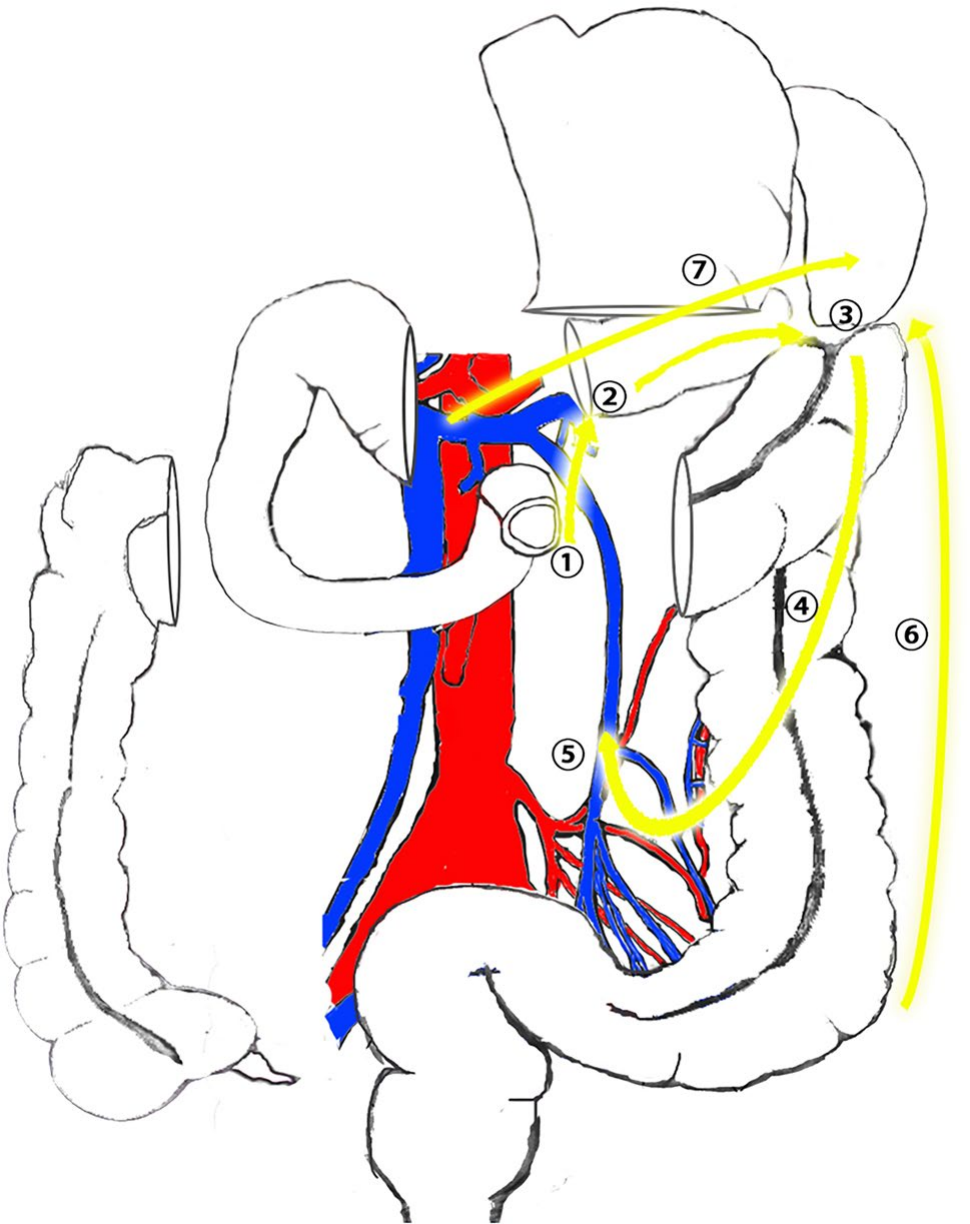
Operation schematic diagram

The patient was placed in the supine position, with legs separated by 45–60°. After the induction of general anesthesia, carbon dioxide was utilized to establish pneumoperitoneum. The arrangement of the trocars and the positioning of the surgeons at different steps are illustrated in Fig. 1. Surgical procedure is illustrated in Fig. 2.

**Fig. 3:**
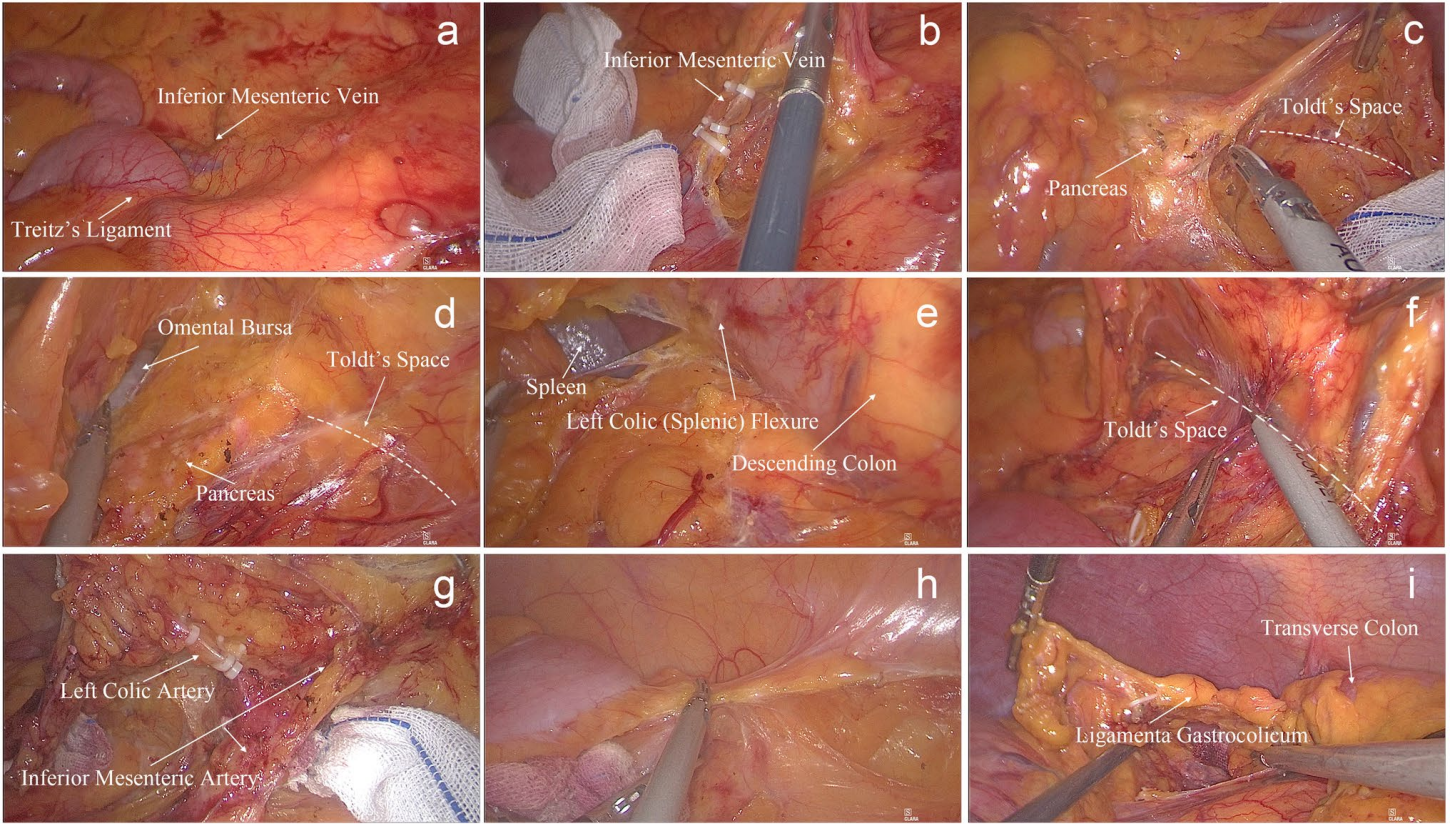
Surgical Procedure of C-shaped Group. **a** Locating Treitz’s ligament and inferior mesenteric vein. **b** entering Toldt’s space. **c** Entering anterior pancreatic space. **d** Expanding Toldt’s space and opening lesser omentum. **e** Dissecting mesentery of the splenic flexure. **f** Continuing to expand Toldt’s space in the medial aspect of the descending colon. **g** Dissecting the No.253 lymph nodes and to cut off the blood supply arteries. **h** Dissecting peritoneum between the descending colon and the abdominal wall and splenocolonic ligament. **i** Dissecting ligamenta gastrocolicum

Step 1: The surgeon first exposed and located Treitz’s ligament and IMV first (Fig. 3A). The IMV was then transected, gaining access to Toldt’s space (Fig. 3B).

Step 2: Further expansion of Toldt’s space toward the cranial direction allowed visualization of the lower pancreatic edge, enabling deliberate dissection of the pancreatic dorsal membrane. Then, the endoscope was advanced through the anterior pancreatic space (Fig. 3C).

Step 3: The anterior pancreatic space was expanded, and the omental bursa was opened (Fig. 3D). Gauze was placed in the omental bursa for position indication. Toldt’s space was further expanded toward the splenic flexure until the mesocolon of the splenic flexure was dissected and exposed spleen (Fig. 3E). Gauze was then placed to indicate the position of the splenic flexure.

Step 4: Inferolateral expansion of Toldt’s space toward the caudal direction continued until the sigmoid colon was reached (Fig. 3F).

Step 5: Identified the root of the IMA (inferior mesenteric artery). In cases where the tumor was located in the splenic flexure, or upper segment of the descending colon, the LCA (left colic artery) was ligated. For tumors located in the middle or lower segment of the descending colon, both the LCA and SA (sigmoid artery) were ligated, or alternatively, the IMA was ligated at its origin. When lesions were located at the descending sigmoid colon junction, the LCA and SA were ligated (Fig. 3G).

Step 6: The left paracolic gutter peritoneum was incised upward toward the splenic flexure (Fig. 3H). For T4 patients, we excised the tumor-invaded portion of the peritoneum or mesentery to ensure radical resection of the tumor.

Step 7: The gastrocolic ligament was incised at the midpoint of the transverse colon, and the gastrocolic ligament, transverse mesocolon, and phrenicocolic ligament were dissected toward the splenic flexure of the colon (Fig. 3I). The splenic flexure and the resection area of the left hemicolon were completely dissected, achieving complete dissociation.

Detailed surgical procedures are shown in supplement files (Online Resource ESM_1).

### Medial-to-lateral group

The surgical procedure was conducted following the classic medial-to-lateral approach technique [16, 17], which can be summarized as follows: the mesentery of the sigmoid colon was opened to access Toldt’s space. The root of the IMA was identified and separated, and the lymph nodes at its base were dissected. Similar to the C-shaped approach, the feeding artery was cut depending on the tumor location. The IMV was then severed. Continuing upward through Toldt’s space, entry into the anterior pancreatic space occurred at the lower edge of the pancreas, followed by expansion along this space toward the spleen until the splenic flexure mesocolon was opened. Full dissociation of Toldt’s space beneath the descending mesocolon was achieved. The subsequent surgical procedure was consistent with step 6 and step 7 of the C-shaped group.

### Reconstruction of the digestive tract

In both groups, digestive tract reconstruction was conducted extraperitoneally via isoperistaltic end-to-end, end-to-side or side-to-side anastomosis using the stapler (Table 1).

**Table 1:** Demographic data and baseline clinical characteristics of patients

Variables		C-shaped Approach (*n* = 92) *n* (%)	Medial-to-lateral Approach (*n* = 95) *n* (%)	*p* value
Age (Years)		60.51 ± 13.31	61.91 ± 13.47	0.477
Gender	Female	47(51.09)	35(36.84)	0.05
	Male	45(48.91)	60(63.16)	
BMI (Kg/m^2^)		22.85 ± 3.24	23.06 ± 3.61	0.678
Tumor invasion	Tis + T0	3(3.33)	4(4.12)	0.212
	T1	6(6.67)	2(2.06)	
	T2	7(7.78)	5(5.15)	
	T3	60(75.56)	56(57.73)	
	T4	16(6.67)	28(30.93)	
Tumor stage	0	3(3.33)	1(1.03)	0.386
	I	11(12.22)	5(5.15)	
	II	39(42.22)	47(49.48)	
	III	34(37.78)	38(39.18)	
	IV	5(4.44)	4(5.15)	
Tumor location	Splenic flexure of colon	26(28.26)	30(31.58)	< 0.001^*^
	Descending colon	50(54.35)	20(21.05)	
	Descending Sigmoid Colon uunction	16(17.39)	45(47.37)	
Anastomosis	End-to-end	27(29.35)	25(26.32)	0.368
	End-to-side	13(14.13)	21(22.11)	
	Side-to-side	52(56.52)	49(51.57)	

*BMI* body mass index

^*^ *p* < 0.05 as statistical significance

Patients in both groups underwent identical postoperative treatment protocols.

### Follow-up

The patients were followed up at regular intervals of 1 month for a duration of 3 months post-surgery to evaluate potential early complications. The patients were asked to complete regular examinations of serum tumor markers, chest and abdominal enhanced computed tomography scans, pelvic magnetic resonance imaging scans, endoscopies, and other related examinations.

### Statistical methods

The normality of continuous variables was initially assessed using the Shapiro‒Wilk test and histograms. For normally distributed data, the t test was employed, whereas for non-normally distributed data, the Mann‒Whitney test was utilized. Categorical variables or ranked data were analyzed via the Chi-square test. Specifically, Pearson’s Chi-square test was used when all cells had an expected count greater than 5; otherwise, Fisher’s exact test was used. In cases where multiple independent variables influenced the normal distribution of the dependent variable, multiple linear regression analysis was conducted to determine statistical significance. The intra-group differences among various subgroups within a unified population were analyzed through ANOVA and subsequent post hoc multiple comparisons, with LSD (Least Significant Difference Method) as the comparison method. An inspection level of *α* = 0.05 and *p* < 0.05 was considered statistically significant. SPSS 23.0 (IBM, Armonk, NY, USA) was used for statistical analysis of the data.

## Results

### Demographic data and baseline clinical characteristics

The study enrolled a total of 187 patients, including 92 patients in the C-shaped group and 95 patients in the Medial-to-Lateral group. The overall cohort included 105 males (56.15%) and 82 females (43.85%). The ages ranged from 25 to 85 years, with an average age of 61.22 ± 13.73 years. All patients are Asian Chinese. The two groups exhibited significant differences in terms of tumor location (*p* < 0.001). There were no statistically significant differences between the two groups in terms of sex, age, BMI, tumor invasion, tumor stage or anastomosis at baseline (*p* > 0.05) (Table 1). The laparoscopic surgery was still performed for certain patients with T4 due to the tumor’s limited invasion range and the potential for radical tumor resection.

### The C-shaped surgical procedure has reliable surgical quality

The number of retrieved lymph nodes, CME rate, and R0 resection rate are crucial indicators for assessing surgical quality. There was no statistically significant difference in the number of retrieved lymph nodes between the two groups (15.55 ± 7.52 vs. 16.11 ± 8.96, *p* = 0.65). Both groups achieved a 100% rate of CME and R0 resection. Therefore, the pancreas-guided C-shaped surgical procedure demonstrated reliable surgical quality.

### The C-shaped surgical procedure offers enhanced safety

We conducted further analysis of surgical safety indices. The C-shaped group experienced lower estimated blood loss (50(20) mL vs. 50(50) mL, *p* = 0.002). To eliminate any potential interference from tumor invasion, tumor location or BMI on total operative time, we employed multiple linear regression analysis to analyze the data for both groups. The results indicated that after excluding any influence, the C-shaped group demonstrated a shorter total operative time than the Medial-to-lateral group did (252.65 ± 50.43 min vs. 280.12 ± 70.45 min, *p* = 0.004), with BMI showing a significant intervention effect on total operative time (*p* = 0.005) (Table 2, Online Resource ESM_2 Table S1). The tumor invasion and tumor location did not affect the overall operative time. There were no significant differences in the time to first flatus, first semi-fluid diet, length of postoperative hospital stay, or incidence of postoperative complications between the two groups. Five cases of splenic injury and three cases of pancreatic injury occurred in Medial-to-lateral approach group. But there was no significant differences between two groups (Table 2).

**Table 2:** Postoperative early outcome and complications analysis

Variables		C-shaped approach (*n* = 92)	Medial-to-lateral approach (*n* = 95)	*p* value
Total operative time (min), mean ± SD		252.65 ± 50.43	280.12 ± 70.45	0.004*
Estimated blood loss (mL), Median(Interquartile Range),		50(20)	50(50)	0.002*
Time to first flatus (days), Median(Interquartile Range)		3(1)	3(1)	0.59
Time to first semi-fluid diet (days), Median(Interquartile Range)		5(1)	5(1.5)	0.11
Postoperative hospital stay (days), mean ± SD		9.59 ± 4.03	8.61 ± 2.82	0.057
Postoperative complications	Ileus, *n* (%)	7(7.61)	3(3.16)	0.946
	Wound infection, *n* (%)	1(1.09)	2(2.11)	
	Pulmonary infection, *n* (%)	0(0)	2(2.11)	
	Anastomotic leakage, *n* (%)	0(0)	1(1.05)	
Intraoperative splenic injury, *n* (%)		0(0)	5(5.26)	0.059
Intraoperative pancreatic injury, *n* (%)		0(0)	3(3.16)	0.246

*SD* standard deviation

^*^ *p* < 0.05 as statistical significance

### The C-shaped surgical procedure is safer and more efficient in obese patients

**Fig. 4:**
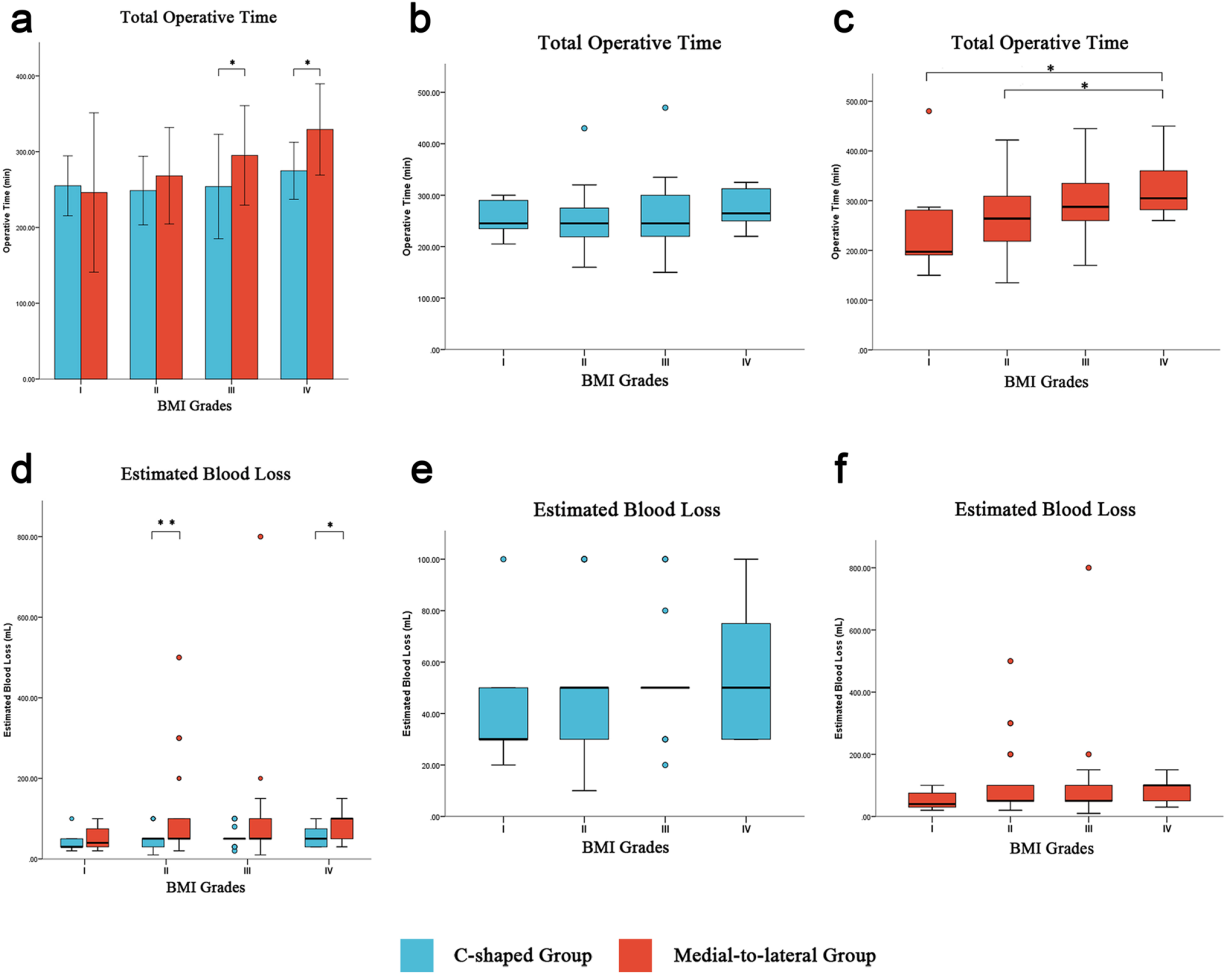
Surgical safety and efficiency analysis of the pancreas-guided C-shaped surgical procedure. **a** and** d** Difference analysis of surgical safety and efficiency between two groups based on BMI grades. **b** and** e** Intra-group difference analysis of C-shaped Group. **c** and** f** Intra-group difference analysis of Medial-to-lateral Group. BMI: Body Mass Index, * *p* < 0.05, the data were statistically different

To further investigate the safety and efficiency of new surgical procedures in obese patients, we classified all patients into four categories according to the obesity grading standards of the Chinese population [18] (Grade I: BMI < 18.5 kg/m^2^, underweight; Grade II: 18.5 ≤ BMI < 24 kg/m^2^, normal weight; Grade III: 24 ≤ BMI < 28 kg/m^2^, overweight; Grade IV: BMI ≥ 28 kg/m^2^, obesity). The two groups exhibited no difference in terms of BMI grade distribution (*p* = 0.675) (Table 3). We compared differences in surgical safety and efficiency indices among patients in different BMI grades. The results revealed that patients with higher BMIs (grades III-IV) in the C-shaped group had shorter total operative time than Medial-to-lateral group. In addition, among patients with grade II and IV BMI, those in the C-shaped group experienced less estimated blood loss (Fig. 4, Table 3). But the indices, including time to first flatus, time to first semi-fluid diet, length of postoperative hospital stay and incidence of postoperative complications, did not exhibit significant differences (Table 3).

**Table 3:** Effect of BMI grades on Surgical safety and efficiency indices between two groups

Variables	BMI grades	C-shaped *n* = 92	Medial-to-lateral *n* = 95	*p* value
Patient Count n(%)	I	5(5.43)	8(8.42)	0.675
	II	57(61.96)	51(53.68)	
	III	22(23.91)	26(27.37)	
	IV	8(8.7)	10(10.53)	
Total operative time (min) Median(interquartile range)	I	245(75)	197.5(93.5)	0.188
	II	245(57.5)	264(93)	0.073
	III	245(84.5)	287.5(80)	0.041*
	IV	264.5(56.25)	305(88.75)	0.036*
Estimated blood loss (mL) Median(interquartile range)	I	30(50)	40(57.5)	0.703
	II	50(20)	50(60)	0.006*
	III	50(0)	50(55)	0.453
	IV	50(57.5)	100(62.5)	0.045*
First flatus (days) Median(interquartile range)	I	3(2)	3.5(1.75)	0.485
	II	3(1)	3(1)	0.859
	III	3(1)	3(1)	0.402
	IV	4(0.75)	4(1)	0.489
First semi-fluid diet (days) Median(interquartile range)	I	5(2)	5(5)	0.765
	II	5(1.5)	5(1)	0.339
	III	5(1.5)	5(2)	0.226
	IV	6(1.5)	5(1)	0.451
Postoperative hospital stay (days) mean ± SD	I	8(4.5)	8.5(4.75)	0.552
	II	8(3)	8(2)	0.259
	III	8(2.5)	9(2)	0.482
	IV	10.5(10.75)	8(2.25)	0.242
Postoperative complications n(%)	I	1	2	/
	II	4	4	
	III	1	0	
	IV	2	2	

*BMI* body mass index, *SD* standard deviation

^*^ *p* < 0.05 as statistical significance

Further, we compared surgical safety indices among patients with different BMI grades within the two groups. The results suggest that the total operative time for obese patients in Medial-to-lateral group was longer compared to normal weight patients (Fig. 4, Online Resource ESM_2 Table S2), while there were no significant differences observed among patients with different BMI grades in the C-shaped group. The postoperative hospital stay for patients in C-shaped group with BMI grade IV was longer compared to those with BMI grade II (Fig. 4, Online Resource ESM_2 Table S3). Therefore, laparoscopic left hemicolectomy via the pancreas-guided C-shaped surgical procedure has superior advantages for obese patients.

### Learning curve analysis for the C-shaped surgical procedure

**Fig. 5:**
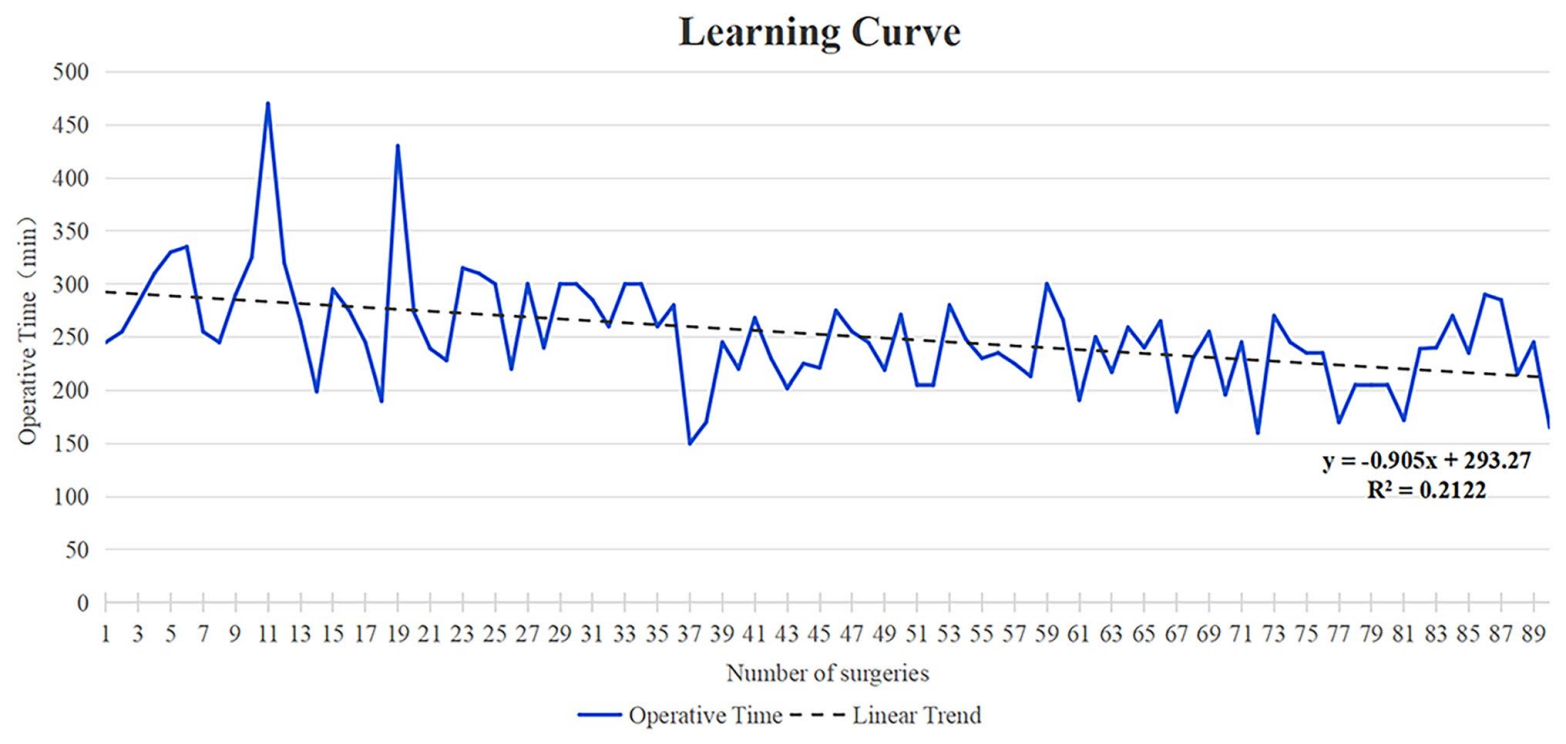
Learning curve of the pancreas-guided C-shaped surgical procedure

The procedure in the C-shaped group was consistently performed by the same surgeon, thus enabling a comprehensive analysis of the learning curve associated with total operative time. With an increasing cumulative number of operations, a gradual decline in the surgeon’s total operative time was observed. Notably, after 20 operations were completed, the average total operative time exhibited a gradual decrease, with the fluctuation converging toward stability (242.2 ± 39.62 min) (Fig. 5). Consequently, our findings indicate that the pancreas-guided C-shaped surgical procedure results in a shorter learning curve and facilitates quicker mastery by surgeons.

## Discussion

Obesity is considered a significant risk factor for laparoscopic left hemicolectomy due to the presence of hypertrophic mesentery and omentum, which significantly impede intra-abdominal space and hinder complete exposure of the surgical area. Furthermore, the hypertrophic mesentery and peritoneum contribute to an unclear anatomic plane, increasing the likelihood of entering incorrect space and potentially causing damage to blood vessels and organs. A clinical study involving 71,084 patients demonstrated that obese patients exhibited a heightened prevalence of surgical complications within 30 days post-surgery, an extended postoperative hospital stay, and an elevated readmission rate [4]. Additional clinical studies have also indicated that obese patients experience increased intraoperative blood loss [3] and face a greater risk of postoperative wound infection (7–8%), anastomotic leakage(2–7%) and ileus (3–8%) [19–23]. The augmented surgical complexity in obese patients results in a notably elevated conversion rate, particularly for novice practitioners, compared to individuals with normal weight [24–26]. Therefore, it is imperative to provide obese patients with a laparoscopic colectomy procedure that ensures both enhanced safety and efficacy.

When patients presents with obesity [3], obstructive colon cancer [10], tumor invasion of surrounding tissues [9], The incidence of pancreatic injury, splenic injury, and ureteral injuries were 0.6%, 6%, and 0.7–1% respectively during the mobilization of splenic flexure [7, 27, 28]. Toldt’s space is a crucial anatomic plane for separation of the mesocolon and complete mesocolic excision. However, it’s a challenge to precisely access Toldt’s space in obese patients. In addition, it is crucial to accurately identify the pancreas and prevent injury to the blood vessels supplying the spleen. However, in obese patients, the presence of a hypertrophic mesentery and subperitoneal fat layer poses challenges in accurate identification. Particularly during dissociation of the splenic flexure mesentery, it is common for the splenic vessels concealed within the splenocolic ligament to be inaccurately identified, resulting in potential injury, bleeding, and ischemia of the spleen. To mitigate this risk, alternative techniques, such as inferior pancreatic approach [14], cranial-to-caudal approach [10], complete medial approach [11], “four-step” methods [12], combined approach [29], have been developed. Preliminary evidence suggests that these novel surgical approaches help circumvent certain operative hazards. However, there are still some important shortcomings, such as the complexity of the surgical procedure, the lack of technical applicability, and the high difficulty of the operation.

The inferior pancreatic approach reported by T. Kumamoto [14], treitz ligament-guided medial approach [30] and medial approach [15, 31] demonstrated that utilizing Treitz’s ligament and adjacent IMV as positioning markers facilitate easier access to Toldt’s space. Cranial-to-caudal approach [10], bursa omentalis approach [13], complete medial approach [11] and pancreas-guided approach reported by He Ziyan [32] emphasize that pancreas-guided mobilization of the splenic flexure is a safer and easier method. After incising the dorsal membrane of the pancreas and accessing the anterior pancreatic space, we were able to precisely delineate the contour of the pancreas in order to minimize potential damage, while simultaneously expediting the separation of the transverse mesocolon. Integrating the advantages of these surgical approaches, we adopted the medial approach as the starting step to access the Toldt’s space and extended this space in a pancreas-guided direction. Then we modified the remaining surgical steps. Instead of incising gastrocolic ligament, we continue to fully expand Toldt’s space to the sigmoid colon, thereby facilitating the identification of the inferior mesenteric artery and enabling lymph node dissection with greater ease. After that, we incised the peritoneum of the left paracolic gutter adjacent to the sigmoid colon and dissected toward the splenic flexure. The gastrocolic ligament was incised to complete the mobilization of the splenic flexure at the final step. The optimized procedure operates more smoothly and efficiently, does not require frequent changes in the position of surgeon and assistants. Surgeon will be able to access Toldt’s space and achieve complete mesocolic excision more easily.

The results of our statistical analysis demonstrate that this novel surgical procedure is capable of achieving complete mesocolic excision, conducting thorough lymph node dissection, and ensuring consistent surgical quality. Surgical safety analysis revealed that the C-shaped procedure resulted in a shorter total operative time, reduced intraoperative blood loss. The C-shaped surgical procedure enables the simultaneous completion of most operations, including splenic flexure mobilization and blood vessels ligation, within Toldt’s space. The entire procedures are coherent and smooth, exhibiting a well-defined anatomic structure. The surgeon can perform the operation with greater efficiency and simplicity.

For obese patients with BMI grade III and IV in C-shaped Group, the total operative time was shorter and the estimated blood loss was reduced. Further intra-group analysis revealed that utilizing the medial-to-lateral approach for surgical procedure on obese patients will result in a significant increase in total operative time. Conversely, implementing the C-shaped procedure can ensure that safety and efficiency during surgery for obese patients is equivalent to that of normal weight patients, surpassing the medial-to-lateral approach in all aspects. In addition, the postoperative hospital stay was longer for patients with BMI grade IV compared to those with BMI grade II. We examined the clinical data of all patients, and only eight patients with BMI grade 4, among whom 1 experienced ileus and another had wound infection. Both complications significantly prolonged the duration of hospitalization after surgery. In addition, it is important to note that the limited sample size also influenced the statistical outcomes. Furthermore, after 20 surgeries were completed, there was a significant decrease in the total operative time, indicating that this new procedure has a short learning curve and is easier to master. Moreover, this procedure is applicable for not only colon cancer surgery but also all surgeries involving left hemicolectomy.

The following technical considerations should be noted during the operation: 1. When entering and expanding Toldt’s space, timely identification of the lower margin of the pancreas, along with active opening of the dorsal membrane of the pancreas into the anterior pancreatic space, is crucial. 2. During spatial expansion of the anterior pancreatic space, it is necessary to open the omental bursa and place gauze at both ends (omental bursa and splenic flexure) for precise localization, which facilitates subsequent steps. 3. It is necessary to directly incise the splenic flexure mesocolon in step 3 and fully extend Toldt’s space in step 4. These measures enhance efficiency and safety during steps 6 and 7 while preventing spleen injury.

Owing to the limitations of analytical methods and the size of the institution where the study was conducted, there are still some shortcomings in this study. First, it was not possible to retrieve specific operation times for the in vivo part of the operation from previous surgical records; therefore, this study analyzed only the total operative time rather than individual in vivo operation times. Second, a retrospective analysis approach was employed, and various statistical analysis methods were utilized to mitigate confounding factor interference with the final results. However, despite these efforts, the sample size was insufficient, necessitating prospective multi-center clinical studies for a comprehensive evaluation of surgical quality and safety associated with this surgical approach.

## Conclusion

The pancreas-guided C-shaped surgical procedure for laparoscopic left hemicolectomy in obese patients ensures smooth surgical procedure with consistent high-quality outcomes and enhanced safety. Moreover, this surgical procedure has a short learning curve, enabling easier mastery and widespread adoption in the field of laparoscopic left hemicolectomy.

## Supplementary Information

Below is the link to the electronic supplementary material.

Supplementary file1 (MP4 137451 KB)

Supplementary file2 (PDF 61 KB)

## Data Availability

The datasets generated during the current study are available in the Science Data Bank repository, 10.57760/sciencedb.16845.
