# Supplementary material for: Pancreas-guided C-shaped surgical procedure: a safer and more efficient procedure for laparoscopic left hemicolectomy in obese patients
Source: Updates Surg. 2025 Jan 18;77(2):343–53. doi: 10.1007/s13304-025-02071-x (PMC11961493; doi:10.1007/s13304-025-02071-x)
Supplement: Supplementary file 2 — Supplementary file2 (PDF 61 KB) [file 13304_2025_2071_MOESM2_ESM.pdf]

Supplementary Files

Article Title: Pancreas-guided C-shaped Surgical Procedure: a Safer and More Efficient Procedure for Laparoscopic Left Hemicolectomy in Obese Patients

Journal: Updates in Surgery

Authors' names: Huaqi Zhang [1*]; Sen Wang [1*]; Zhensheng Chen [1]; Tedong Luo [1]; Jinpeng Cao[1]; Zhicheng Li [1]; Yong Ji [1]

Authors' affiliations:

1 Department of Gastrointestinal surgery, the First People's Hospital of Foshan, No. 81 Lingnan Avenue North, Foshan, China

*: These authors contributed equally to this study

Corresponding author :

Yong Ji

Address: The First People's Hospital of Foshan, No. 81 Lingnan Avenue North, Foshan, China; Post code:528000; City: Foshann; Country: China

Telephone: 86-757-83161619

Fax: 86-757-83812566

Email: jyongfsyyy@sina. Com
ORCID: 0009-0006-6429-6500

Table S1 Multiple Linear Regression Analysis of Total Operative Time

| Factor | Unstandardized Coefficients | | Standardized Coefficients | t | significant | 95.0% Confidence Interval for B | |
|---|---|---|---|---|---|---|---|
| | B | Std. Error | Beta | | | Lower Bound | Upper Bound |
| Constant | 138.510 | 42.009 | | 3.297 | .001* | 55.624 | 221.397 |
| Surgical Procedures | 33.204 | 9.755 | .243 | 3.404 | .001* | 13.956 | 52.452 |
| BMI | 3.123 | 1.424 | .156 | 2.194 | .030* | .314 | 5.932 |
| Tumor Invasion | 2.818 | 5.446 | .037 | .517 | .605 | -7.928 | 13.563 |
| Tumor Location | .753 | 6.986 | .008 | .108 | .914 | -13.032 | 14.537 |

BMI: Body Mass Index

*: $p<0.05$ as statistical significance

Table S2 Intra-group difference analysis of C-shaped Group based on BMI grades

| Dependent Variables | BMI Grades | | Mean Difference | Std. Error | p value | 95% Confidence Interval | |
|---|---|---|---|---|---|---|---|
| | | | | | | Lower Bound | Upper Bound |
| Total operative time | I | II | 6.3158 | 23.6517 | 0.7901 | -40.6871 | 53.3186 |
| | | III | 0.9091 | 25.1232 | 0.9712 | -49.0180 | 50.8362 |
| | | IV | -20.5000 | 28.9089 | 0.4801 | -77.9503 | 36.9503 |
| | II | III | -5.4067 | 12.7278 | 0.6720 | -30.7006 | 19.8872 |
| | | IV | -26.8158 | 19.1454 | 0.1648 | -64.8632 | 11.2316 |
| | III | IV | -21.4091 | 20.9360 | 0.3093 | -63.0150 | 20.1968 |
| Estimated blood loss | I | II | -2.4211 | 11.36077 | 0.832 | -24.9982 | 20.1561 |
| | | III | -8.0909 | 12.06757 | 0.504 | -32.0727 | 15.8909 |
| | | IV | -9.0000 | 13.88596 | 0.519 | -36.5954 | 18.5954 |
| | II | III | -5.6699 | 6.11363 | 0.356 | -17.8194 | 6.4797 |
| | | IV | -6.5789 | 9.19620 | 0.476 | -24.8545 | 11.6966 |
| | III | IV | -.9091 | 10.05631 | 0.928 | -20.8939 | 19.0757 |
| First flatus | I | II | -.4947 | .93384 | 0.598 | -2.3505 | 1.3611 |
| | | III | -.1909 | .99194 | 0.848 | -2.1622 | 1.7804 |
| | | IV | -.4750 | 1.14141 | 0.678 | -2.7433 | 1.7933 |
| | II | III | .3038 | .50253 | 0.547 | -.6948 | 1.3025 |
| | | IV | .0197 | .75592 | 0.979 | -1.4825 | 1.5220 |
| | III | IV | -.2841 | .82662 | 0.732 | -1.9268 | 1.3586 |
| First semi-fluid diet | I | II | -.2667 | 1.02989 | 0.796 | -2.3134 | 1.7800 |
| | | III | -.0091 | 1.09396 | 0.993 | -2.1831 | 2.1649 |
| | | IV | -.2250 | 1.25881 | 0.859 | -2.7266 | 2.2766 |
| | II | III | .2576 | .55422 | 0.643 | -.8438 | 1.3590 |
| | | IV | .0417 | .83367 | 0.960 | -1.6151 | 1.6984 |
| | III | IV | -.2159 | .91164 | 0.813 | -2.0276 | 1.5958 |
| Postoperative hospital stay | I | II | -.5930 | 1.85766 | 0.750 | -4.2847 | 3.0987 |
| | | III | -1.1727 | 1.97323 | 0.554 | -5.0941 | 2.7487 |
| | | IV | -3.9000 | 2.27056 | 0.089 | -8.4123 | .6123 |
| | II | III | -.5797 | .99967 | 0.563 | -2.5664 | 1.4069 |
| | | IV | -3.3070 | 1.50372 | 0.030* | -6.2953 | -.3187 |
| | III | IV | -2.7273 | 1.64436 | 0.101 | -5.9951 | .5405 |

*: $p<0.05$ as statistical significance

Table S3 Intra-group difference analysis of Medial-to-lateral Group based on BMI grades

| Dependent Variables | BMI Grades | | Mean Difference | Std. Error | p value | 95% Confidence Interval | |
|---|---|---|---|---|---|---|---|
| | | | | | | Lower Bound | Upper Bound |
| Total operative time | I | II | -22.0319 | 25.83327 | 0.396 | -73.3465 | 29.2828 |
| | | III | -48.9904 | 27.46574 | 0.078 | -103.5477 | 5.5669 |
| | | IV | -83.1750 | 32.22365 | 0.011* | -147.1833 | -19.1667 |
| | II | III | -26.9585 | 16.37032 | 0.103 | -59.4761 | 5.5591 |
| | | IV | -61.1431 | 23.49434 | 0.011* | -107.8118 | -14.4745 |
| | III | IV | -34.1846 | 25.27831 | 0.180 | -84.3969 | 16.0276 |
| Estimated blood loss | I | II | -33.2598 | 38.88098 | 0.395 | -110.4921 | 43.9725 |
| | | III | -45.6731 | 41.33796 | 0.272 | -127.7859 | 36.4397 |
| | | IV | -41.7500 | 48.49897 | 0.392 | -138.0872 | 54.5872 |
| | II | III | -12.4133 | 24.63853 | 0.616 | -61.3547 | 36.5281 |
| | | IV | -8.4902 | 35.36072 | 0.811 | -78.7299 | 61.7495 |
| | III | IV | 3.9231 | 38.04572 | 0.918 | -71.6501 | 79.4962 |
| First flatus | I | II | .4240 | .34703 | 0.225 | -.2653 | 1.1134 |
| | | III | .5288 | .36896 | 0.155 | -.2041 | 1.2617 |
| | | IV | .2750 | .43288 | 0.527 | -.5849 | 1.1349 |
| | II | III | .1048 | .21991 | 0.635 | -.3320 | .5417 |
| | | IV | -.1490 | .31561 | 0.638 | -.7759 | .4779 |
| | III | IV | -.2538 | .33958 | 0.457 | -.9284 | .4207 |
| First semi-fluid diet | I | II | .4020 | .60028 | 0.505 | -.7904 | 1.5943 |
| | | III | .5769 | .63821 | 0.368 | -.6908 | 1.8447 |
| | | IV | .1000 | .74877 | 0.894 | -1.3873 | 1.5873 |
| | II | III | .1750 | .38039 | 0.647 | -.5806 | .9306 |
| | | IV | -.3020 | .54593 | 0.582 | -1.3864 | .7825 |
| | III | IV | -.4769 | .58739 | 0.419 | -1.6437 | .6898 |
| Postoperative hospital stay | I | II | 1.7721 | 1.06521 | 0.100 | -.3439 | 3.8880 |
| | | III | 1.8558 | 1.13252 | 0.105 | -.3939 | 4.1054 |
| | | IV | .5250 | 1.32871 | 0.694 | -2.1143 | 3.1643 |
| | II | III | .0837 | .67501 | 0.902 | -1.2571 | 1.4245 |
| | | IV | -1.2471 | .96877 | 0.201 | -3.1714 | .6773 |
| | III | IV | -1.3308 | 1.04233 | 0.205 | -3.4012 | .7397 |

*: $p<0.05$ as statistical significance
